# Longitudinal Morphomolecular Monitoring of Head and Neck Carcinogenesis

**DOI:** 10.1002/advs.202522344

**Published:** 2026-09-30

**Authors:** Jianrong Qiu, Priyanka G. Bhosale, Vishal Kumar, Jeyrroy Gabriel, Shiyue Liu, Ciro Chiappini, Richard J. Cook, Mads S. Bergholt

**Affiliations:** ^1^ Centre for Craniofacial & Regenerative Biology King's College London London UK; ^2^ Department of Haematology University College London Cancer Institute London UK; ^3^ CGRM, BMBS, Guy's Campus King's College London London UK; ^4^ St. John's Histopathology Tissue Sciences Synnovis Analytics St. Thomas’ Hospital London UK; ^5^ Centre For Oral Clinical and Translational Sciences King's College London London UK

**Keywords:** artificial intelligence, endoscopy, fiber‐optics, optical coherence tomography, raman spectroscopy

## Abstract

Current diagnostic approaches for head and neck cancer rely primarily on white‐light endoscopy and tissue biopsy, providing only static, episodic assessments that fail to capture dynamic changes in tumor biology over time. The absence of continuous, detailed molecular and microscopic structural information makes early identification of dysplasia or cancer difficult and can delay critical treatment. Here, we introduce a unified, label‐free endoscopic platform that combines Raman spectroscopy (RS) and optical coherence tomography (OCT) within a miniaturized, forward‐viewing fiber‐optic probe. This integration enables simultaneous video‐rate OCT imaging and 1 s RS acquisition, linking morphological and molecular tissue trajectories in vivo. We developed an explainable AI‐based fusion framework that learns joint temporal representations of biochemical and structural features to classify tissue states while highlighting the most influential predictors. In a longitudinal 4NQO‐induced murine model of carcinogenesis, the system tracked epithelial transformation from hyperplasia through dysplasia and carcinoma in situ to invasive cancer. OCT provides the dominant structural contrast in this thin‐epithelium murine model, while RS adds complementary molecular specificity. By capturing temporally resolved morphological and molecular trajectories in vivo, this platform demonstrates a new approach for early cancer detection and has potential to support surveillance and clinical decision‐making.

## Graphical abstract

A miniaturized endoscopic probe that integrates Raman spectroscopy and optical coherence tomography, enabling simultaneous molecular and structural imaging of living tissue, was developed. Applied longitudinally in a mouse model of head and neck cancer, the system tracks disease progression from precancerous lesions to invasive cancer. An explainable AI framework accurately classifies tissue states, linking biochemical and structural changes.

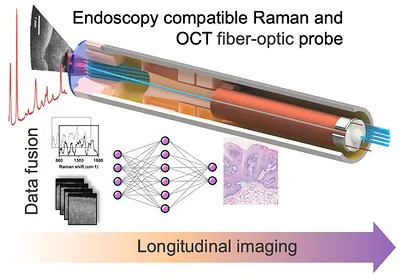

## 1 Introduction

Head and neck squamous cell carcinoma (HNSCC) remains a global health challenge, imposing substantial health, social, and economic burdens [1]. Despite advances in treatment, the prognosis for HNSCC remains poor, as the majority of cases are diagnosed at advanced stages. This makes early detection crucial, as it creates a window of opportunity for timely disease management, better clinical outcomes, and the preservation of vital functions such as speech and swallowing, ultimately enhancing patients’ quality of life. Some precancerous lesions remain latent without ever progressing, while others (10%–25%) are more likely to advance to malignancy or develop resistance to treatment [2, 3, 4]. Leaving high‐risk lesions in situ leads to evolution of the tumor microenvironment and exacerbation of disease, with devastating consequences for the patient. Therefore, monitoring the disease trajectory of lesions is of significant clinical importance to initiate targeted and aggressive treatment (e.g., resection, ablation, or neoadjuvant chemotherapy). Conventional endoscopic diagnostic techniques are based on visual examination using white light endoscopy or narrowband endoscopy. These techniques only offer gross morphological assessment. When combined with biopsy and histopathology, this approach is inherently invasive and hinders continuous longitudinal monitoring due to the need for repeated tissue sampling. Medical imaging, including computed tomography, magnetic resonance imaging, and positron emission tomography, offers 3D monitoring of disease, although with limited spatial resolution and molecular specificity.

HNSCC is a multistep disease driven by alterations in genomic, cellular, molecular, and tissue microarchitecture [5]. The diverse oncogenomic landscape largely stems from the selection and clonal expansion of tumor‐initiating cells that acquire various molecular and genetic alterations [6]. This includes frequent mutations of tumor suppressor genes such as *TP53*, *FAT1*, *NOTCH1*, *PIK3CA*, and *CASP8*. Many of these mutations disrupt cellular processes that regulate proliferation and differentiation, resulting in a progressively altered molecular profile within the tissue [7]. Concurrently, these cellular and molecular changes are accompanied by microscopic morphological transformations. Dysplastic tissues often exhibit loss of epithelial cohesion, disrupted stratification, basal cell hyperplasia, epithelial thickening, and irregularly shaped rete pegs. These structural abnormalities are strongly associated with malignant transformation and increased risk of lesion recurrence [8]. As dysplasia advances to carcinoma, tumor‐initiating cells invade the surrounding stroma, breaking down normal structural boundaries and leading to highly disorganized tissue architecture.

Fiber‐based optical imaging techniques have emerged as promising tools, offering a unique combination of label‐free, non‐invasive modalities that deliver high‐resolution morphological imaging alongside molecular analysis, paving the way for more dynamic and informative tissue assessment. Techniques such as Raman spectroscopy (RS) [9, 10, 11], optical coherence tomography (OCT), confocal laser endomicroscopy [12], and multiphoton microscopy [13, 14, 15], including second harmonic generation, two‐photon excited fluorescence, and coherent anti‐Stokes Raman spectroscopy, have demonstrated considerable potential for cancer diagnostics. Multimodal combinations have also been explored [16, 17, 18, 19]. RS exploits the inelastic scattering of light to provide detailed vibrational signatures of molecular bonds, thereby enabling the identification of biomolecular fingerprints and disease markers. RS provides rich molecular fingerprints of tissue pathology yet is blind to the morphological context in which these changes occur. OCT addresses this gap by offering high‐resolution, cross‐sectional reconstructions of tissue microarchitecture, enabling the visualization of subtle structural perturbations associated with disease onset and progression [20].

Integrating RS and OCT into a single, miniaturized endoscopic probe presents major engineering challenges. RS depends on efficient photon collection and distal optical filtering. Meanwhile, OCT relies on beam scanning and integrated focusing optics to generate high‐resolution images. These fundamentally different optical demands create a complex set of design trade‐offs, making it difficult to merge both modalities into one compact fiber‐optic probe compatible with endoscope instrument channels. Another critical challenge is balancing a sufficient optical field of view (FOV) with the strict mechanical constraints required for miniaturization within standard endoscopic channels. Historically, pioneering efforts to combine RS and OCT in a fiber‐optic format have therefore been limited to large or side‐viewing designs with dimensions (at centimeter scales) that exceed requirements needed in clinical endoscopy [21, 22, 23]. For instance, an oral‐compatible handheld side‐viewing benchtop probe was developed [21], while a coaxial RS and OCT benchtop handheld probe has been introduced for skin analysis [22]. Temporal co‐registration remains another challenge: RS acquires spectra on subsecond timescales, whereas OCT provides video‐rate images. This temporal discrepancy demands novel fusion strategies that can integrate the disparate data into a joint, coherent representation. Achieving true integration within a miniaturized, forward‐viewing fiber‐optic probe that enables real‐time, co‐registered acquisition remains a significant unmet challenge.

Here, we introduce a unified, forward‐viewing endoscopic platform that integrates Raman spectroscopy and optical coherence tomography (RS‐OCT). The system incorporates a miniaturized, high‐throughput fiber‐optic probe with a spliced‐fiber architecture and distal beam scanning. Designed to be compatible with standard clinical endoscopes, we applied this probe in a longitudinal in vivo mouse model of head and neck carcinogenesis. Our approach enables high‐resolution, video‐rate longitudinal OCT imaging of tissue microarchitecture alongside subsecond RS‐based molecular profiling. To interpret the multimodal data, we developed a quantitative analysis pipeline and an explainable deep learning‐based fusion framework that classifies tissue by integrating biomolecular and morphological signatures. Collectively, this technology provides a non‐invasive strategy for comprehensive temporal tissue characterization in internal organs, with translational potential to inform precision diagnostics and guide personalized therapeutic decision‐making in oncology.

## 2 Results

### 2.1 Unified RS‐OCT Fiber‐Optic Endoscopy

We developed a compact, integrated endoscopic platform that unites RS and OCT within single, miniaturized fiber‐optic probes (2.2 and 3.4 mm diameter versions; Figure 1a). RS was implemented with 785 nm laser excitation and high‐throughput signal collection across the 800–1800 cm^−^^1^ fingerprint window, while OCT imaging employed a custom‐built ∼1325 nm spectral‐domain system. This ensures that the Raman fingerprint range (800–1800 cm^−1^ or ∼837–914 nm) does not overlap with the OCT spectral domain and vice versa. The developed miniaturized probe utilized a sandwich‐like fiber configuration: two arrays of RS excitation/collection fibers were angled toward the optical axis (Figure S1), flanking a centrally positioned single‐mode fiber for OCT (Figure 1b). Since RS has limited compatibility with distal micro lenses, the OCT light focusing was achieved by splicing the single‐mode fiber with a no‐core fiber (NCF) and a gradient index fiber (GIF) to focus ∼0.5 mm into the tissue (Figure 1c). This ensured that no parasitic Raman signals were generated from any distal optics. This design enabled OCT B‐scanning across a central opening, providing a 1 mm field of view with axial and transverse resolutions of 10 and ∼25 µm, respectively. By maintaining a consistent spatial relationship between the structural and molecular sensing components, this symmetric design allows for co‐localization of morphological features with their corresponding biomolecular signatures. The internal structure of the fiber probe was 3D printed to house a piezo tube enclosed by biocompatible stainless steel and sealed with a 10‐degree tilted CaF_2_ window to reduce OCT back reflections (Figure 1d).

**FIGURE 1:**
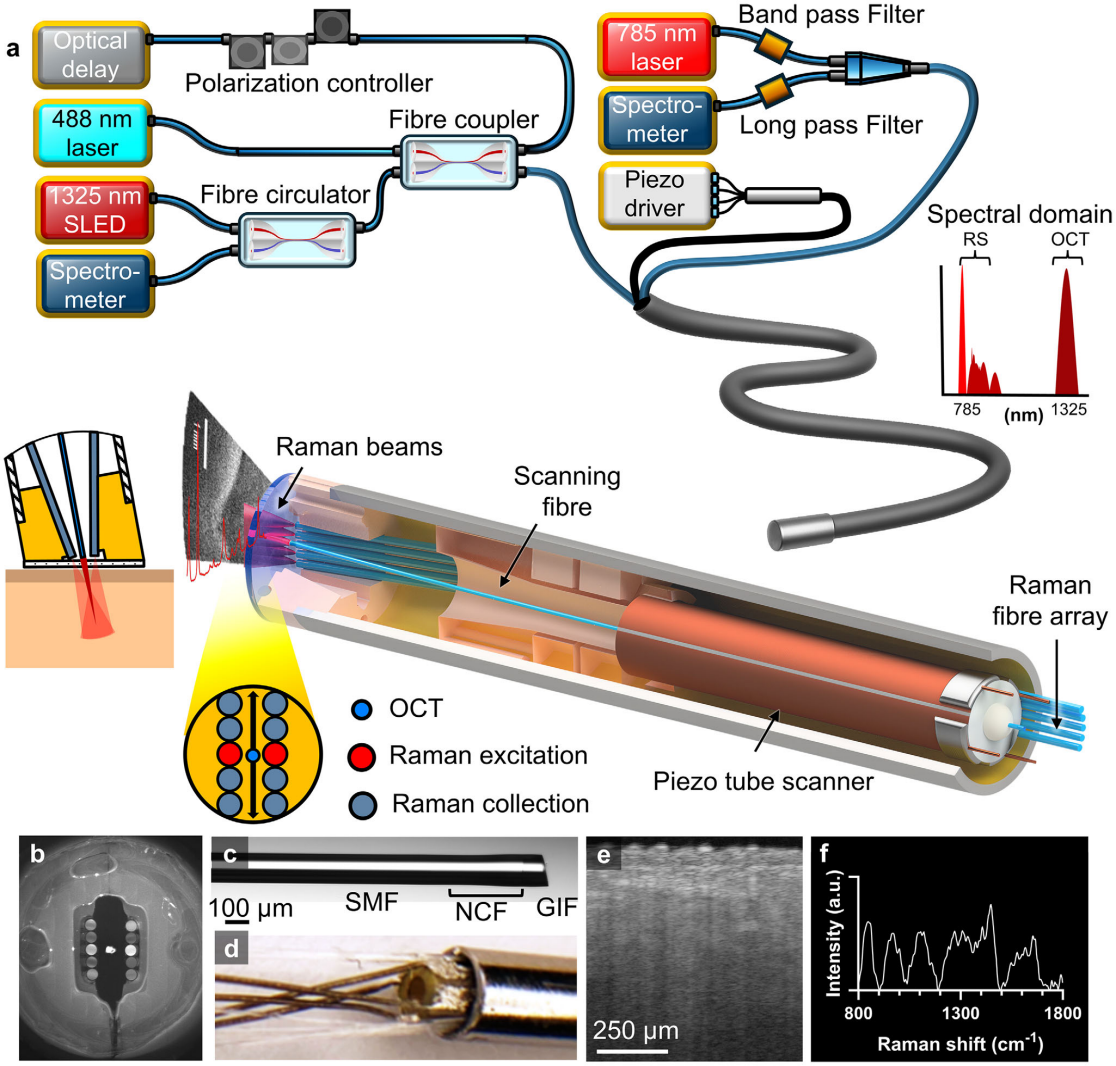
Unified multimodal fiber‐optic probe for Raman spectroscopy (RS) and OCT (a). The multimodal Raman‐optical coherence tomography (RS‐OCT) fiber‐optic probe, designed based on a spectral‐domain OCT scanning platform, utilizes a compact piezoelectric (PZT) tube actuator for beam scanning. The RS component operates with 785 nm laser excitation and incorporates a high‐throughput spectrograph for efficient signal detection. Also shown is the overlap of the OCT field of view and Raman sampling. A 488 nm laser was coupled in for OCT fibre alignment. (b) En face view of the fiber probe, showing the central OCT fiber surrounded by two linear arrays of excitation/collection fibers dedicated to Raman signal acquisition. (c) Detailed structure of the spliced single‐mode fiber (SMF) used in the OCT path, consisting of a segment of SMF for light delivery, followed by a no‐core fiber (NCF) for beam expansion, a gradient‐index fiber (GIF) for focusing, terminated with an angle‐cleaved surface for back‐reflection suppression. (d) Assembled probe showing the fiber bundles and electrical wiring. (e) Representative in vivo OCT imaging demonstrating high‐resolution structural visualization. (f) Corresponding in vivo Raman spectra acquired from the same region, providing complementary molecular information.

Processing real‐time multimodal datasets acquired at different timescales remains a major challenge. We constructed a parallel data acquisition and processing framework (Figure S2) implemented with CPU‐based RS analysis and GPU‐based OCT image processing. The OCT images were reconstructed using background removal, k‐domain linearization, Fast Fourier Transform (FFT), and offset/scaling. Raman spectra were processed using system background subtraction, autofluorescence removal using a polynomial background subtraction, and vector normalization. This integrated framework allows us to measure real‐time colocalized high‐quality OCT images (Figure 1e) and near real‐time tissue Raman spectra (Figure 1f). Temporal co‐registration of RS and OCT data was achieved by synchronizing each 1 s RS acquisition with a randomly selected frame from the corresponding OCT sequence, acquired at 46 frames per second.

### 2.2 In Vivo Imaging in a Murine Model of Head and Neck Carcinogenesis

In a murine model of head and neck carcinogenesis, we demonstrated real‐time, in vivo imaging, providing a new view of the disease process at both molecular and microstructural levels. Oral tissue was used as a test bed to represent an inherently challenging multiphenotypic tissue environment. We employed a 4‐Nitroquinoline N‐oxide (4NQO) oral carcinogenesis mouse model, which is well established for studying disease progression and exhibits molecular and histological changes similar to those seen in human HNSCC [7]. RS‐OCT data were collected longitudinally at multiple predefined sites on the tongue (a single anatomical site) in both the 4NQO‐treated group (*n* = 10) and control group (*n* = 10) at multiple time points (weeks 10, 12, 14, 16, 18, and 20) (Figure 2a and Supplementary video). At the study endpoint (week 22), visual inspection revealed lesions at multiple sites on the tongues of 4NQO‐treated mice (Figure S3). Histological evaluation of these tissues was then performed as the gold standard to determine the ground truth disease outcome (Figures 2b and S4). The lesions were histologically categorized as hyperplasia, dysplasia, carcinoma in situ (CIS), and invasive oral squamous cell carcinoma (OSCC) (Figure 2b). Exposure to 4NQO resulted in the development of multifocal hyperplastic and/or mild dysplastic lesions in 100% of 4NQO‐treated mice, with each mouse presenting with at least four lesions (Figure S3c). Of these, 70%–80% progressed to moderate‐to‐severe dysplasia or CIS, while 40%–60% developed microinvasive or invasive OSCC within 22 weeks. OCT imaging enabled clear visualization of epithelial architecture, with control tissues displaying well‐defined, stratified epithelium (Figure 2c). We observed progressive epithelial thickening in carcinogen‐treated tissues, along with increasing architectural disarray, a hallmark of dysplastic and malignant progression as confirmed by endpoint histopathology (Figure 2b). Co‐registered high‐fidelity Raman spectra exhibited distinct biochemical signatures, including prominent peaks at 1301 cm^−^^1^ (CH_2_ twisting/wagging modes from lipids/proteins), 1450 cm^−^^1^ (CH_2_/CH_3_ bending), and 1650 cm^−^^1^ (Amide I, C═O stretching), aligning with recognized spectral markers of biological tissue (Figure 2d) [9, 24]. RS revealed spectral changes between pathologies, as visualized in average difference spectra ±1 standard deviation (SD) (Figure S5), reflecting underlying biochemical remodeling across disease stages. These RS‐OCT results establish the integrated system as a label‐free, multimodal endoscopic platform for tracking carcinogenesis with both spatial and molecular fidelity.

**FIGURE 2:**
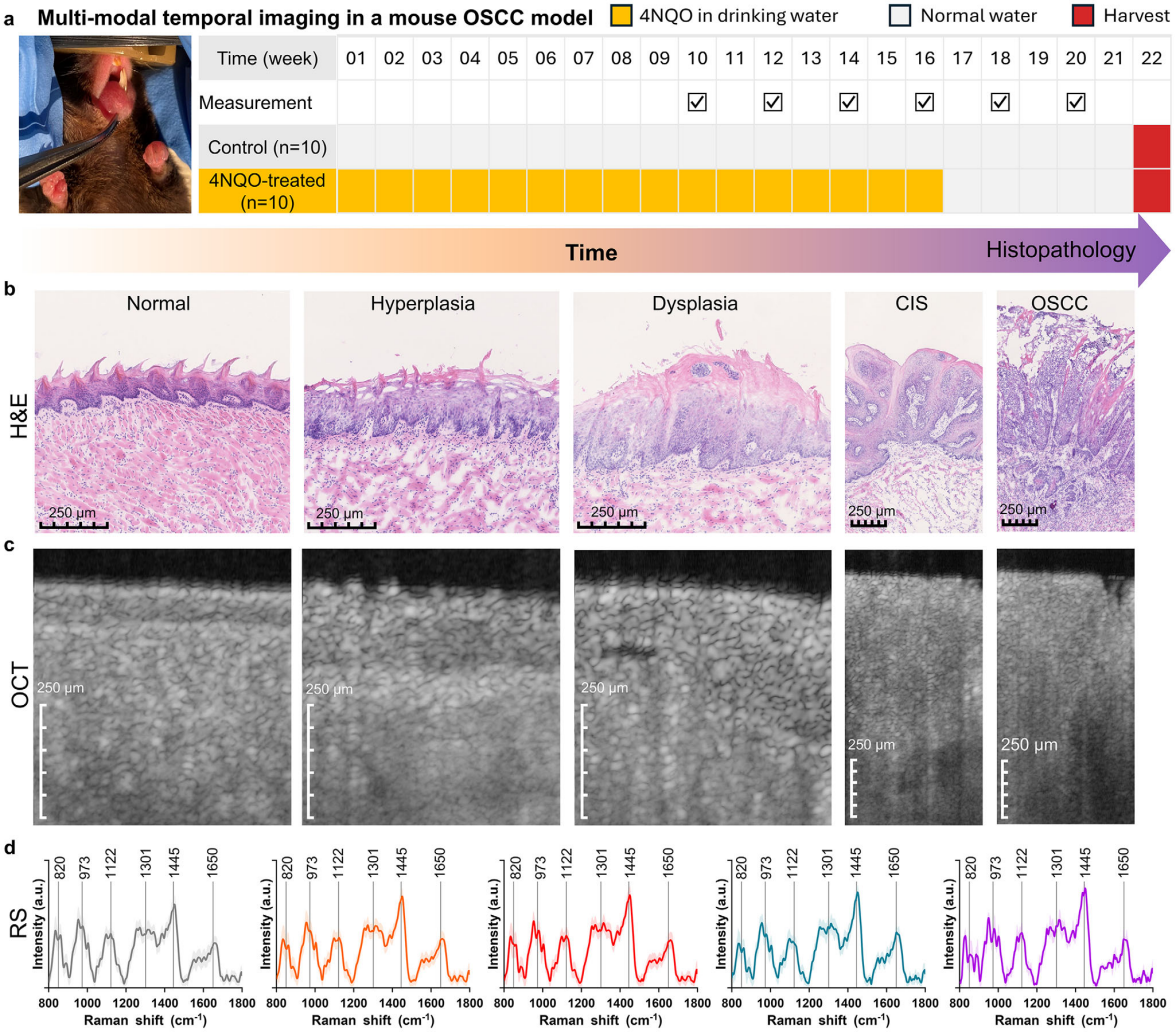
Morphomolecular in vivo monitoring mouse model of head and neck carcinogenesis. (a) Study design: Head and neck carcinogenesis was induced using the chemical carcinogen 4‐Nitroquinoline N‐oxide (4NQO). A cohort of mice (*n* = 10) received 4NQO diluted in drinking water at a concentration of 100 µg/mL for a 16‐week treatment period. Control mice (*n* = 10) received regular water. After the treatment phase, all animals were returned to normal drinking water and monitored until the experimental endpoint at week 22 or until a weight loss exceeding 15% of their peak body weight was observed. RS‐OCT measurements were performed longitudinally at weeks 10, 12, 14, 16, 18, and 20. (b) Representative hematoxylin and eosin (H&E) stained sections from normal, hyperplasia, dysplasia, CIS, and OSCC tissues. (c) In vivo OCT imaging at multiple time points, illustrating the progression from normal epithelium, hyperplasia, to dysplasia, CIS, and OSCC. Imaging captures progressive epithelial thickening, heightened scattering, and increasing tissue disorganization during carcinogenesis. (d) Mean in vivo Raman spectra ±1 standard deviation (SD) corresponding to normal, hyperplasia, dysplasia, CIS, and OSCC tissue, demonstrating subtle molecular alterations throughout disease progression.

### 2.3 Longitudinal Quantitative Profiling of Head and Neck Carcinogenesis

We developed an analytical framework to quantify morphological and molecular changes from multimodal data (see Materials and Methods), with OCT image analysis measuring epithelial thickness and grading stratification and architectural disorganization (Figure 3a). Hyperplastic lesions and lesions with histologically confirmed dysplasia through to CIS/OSCC exhibited progressive epithelial thickening as compared to control tissue (Figure 3b), consistent with histopathological grading (Figure 2b). Both CIS and malignant tissues showed progressive architectural disorganization as the primary morphological indicator (Figure 3c). This could be a result of loss of basal cell polarity together with a breakdown in the orderly maturation and differentiation of epithelial cells, a hallmark of neoplastic transformation. Crucially, our platform captured these dynamic changes noninvasively and without labels. Longitudinal analysis in a single mouse revealed continuous remodeling of epithelial architecture, with quantifiable changes in thickness and stratification over time (Figure 3d), demonstrating the system's capability for real‐time monitoring of disease trajectory.

**FIGURE 3:**
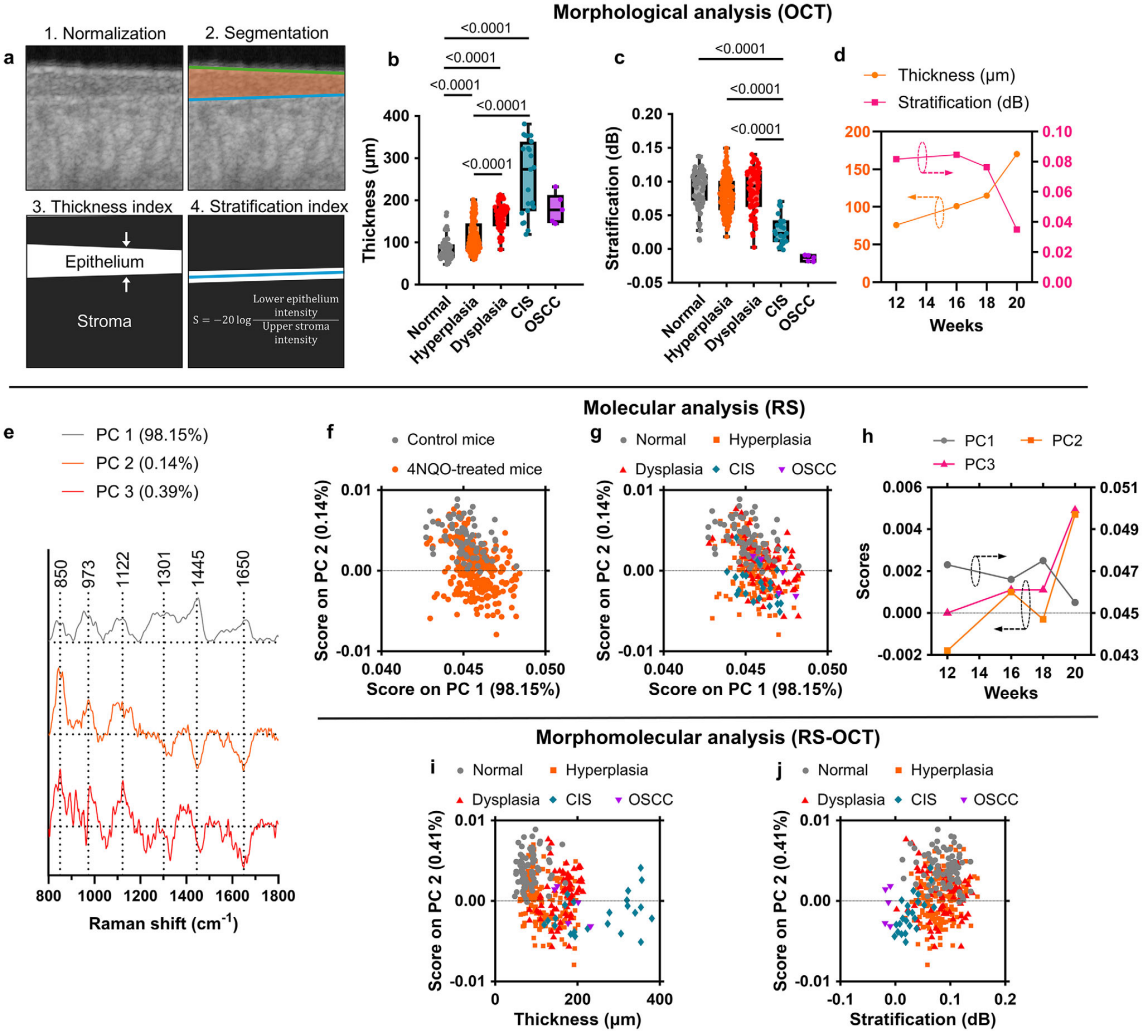
Longitudinal quantitative profiling of head and neck carcinogenesis. (a) Quantitative workflow for optical coherence tomography (OCT) analysis, including epithelial thickness measurement and tissue stratification index calculation. (b) OCT‐derived epithelial thickness measurements for normal, hyperplasia, dysplasia, CIS and OSCC groups. (c) OCT‐based tissue stratification index for normal, hyperplasia, dysplasia, CIS and OSCC groups. (d) Longitudinal monitoring of epithelial thickness and stratification in a single mouse. (e) Principal component analysis (PCA) loadings (PC1, PC2 and PC3). (f) PCA scores (PC1 vs. PC2) from histopathologically confirmed control, hyperplasia, dysplasia, CIS and OSCC tissues. The normal group was well separated from the CIS and OSCC groups. (g) PCA scores (PC1 vs. PC2) for all tissue types. (h) Longitudinal monitoring of molecular changes in a single mouse. (i) Morphomolecular analysis showing the relationship between PC2 and epithelial thickness across tissue states, highlighting how molecular signatures captured by RS correlate with structural changes detected by OCT. (j) Morphomolecular analysis showing the relationship between PC2 and epithelial stratification, illustrating how molecular variation aligns with the degree of tissue organization and differentiation. *p*‐values in (b,c) were calculated by the Wilcoxon rank‐sum test with BH‐FDR correction.

For RS molecular characterization, we applied principal component analysis (PCA) to the Raman spectra, capturing dominant biomolecular variance. PCA loadings highlighted vibrational signatures such as 850 cm^−1^ (collagen), 1301 cm^−1^ (lipids) and 1650 cm^−1^ (lipids/proteins) (Figure 3e). PCA score plots showed separation between control and 4NQO‐treated tissues, reflecting disease‐associated molecular shifts (Figure 3f–g), although with significant overlap. In a single mouse, longitudinal PCA scores revealed a progressive trajectory, in which successive timepoints traced a directional shift along principal components (PCs) associated with specific biochemical changes (Figure 3h). This gradual migration reflected the biochemical remodeling of the tissue microenvironment.

To investigate how molecular alterations relate to morphological remodeling, we correlated the PCA scores from Raman spectra with epithelial thickness and stratification metrics extracted from OCT. Plotting PC2 scores against epithelial thickness revealed improved clustering, in which progressive thickening was accompanied by coordinated shifts in molecular composition (Figure 3i). Similarly, PC stratification plots demonstrated that increasing architectural layering and disorganization tracked with distinct molecular signatures along specific PC axes (Figure 3j). These relationships suggest that the same biochemical pathways captured in the spectral PCs may underlie both hyperproliferative thickening and loss of orderly epithelial structure. The ability to plot molecular states against quantitative OCT imaging metrics highlights the novelty of integrating the two complementary modalities.

### 2.4 AI‐Driven Multimodal RS‐OCT Data Fusion and Classification

We then developed a convolutional neural network (CNN)‐based AI framework to more efficiently extract, fuse, and classify multimodal RS and OCT data (Figure 4a), effectively integrating the complementary strengths of both modalities. In our architecture, each modality was initially processed independently through a dedicated series of convolutional and max‐pooling layers, allowing the network to learn hierarchical, modality‐specific feature representations. The extracted features from the RS and OCT branches were then concatenated and passed through a set of shared fully connected layers to perform feature fusion and joint representation learning. This fusion strategy enabled the model to learn synergistic patterns across compositional and structural domains. As a feasibility demonstration of multimodal data fusion, we benchmarked the classifier using animal‐independent fivefold cross‐validation: in each fold, all measurements from a held‐out set of mice were excluded from training and used only for testing, so that every animal contributing in vivo data was evaluated exactly once as a fully unseen subject. Probabilistic classification of tissue in dysplasia/cancer vs. normal showed an aggregated classification accuracy of 92.3% (fold mean 93.7%, 88.4%–99.0%; 95% confidence interval (CI)), with 92.9% (fold mean 93.1%, 82.9%–100%; 95% CI) sensitivity and 91.8% (fold mean 94.4%, 88.0%–100%; 95% CI) specificity under cross‐validation for RS‐OCT (Figure 4b–d). Given the size of this first in vivo cohort, we present these results as a technological feasibility demonstration of real‐time RS‐OCT fusion rather than as a diagnostic performance claim. A comparison of cross‐validation performance can be found in Figure S7. The fusion network was trained directly on preprocessed Raman spectra and preprocessed OCT B‐scan images. The hand‐engineered morphological metrics (epithelial thickness and stratification index) were used only for the quantitative profiling in Figure 3 and were not provided to the network. Moreover, because cross‐validation withholds animals in turn, every one of the animals served once as a fully unseen test subject, so the aggregated metrics reflect prediction across all animals.

**FIGURE 4:**
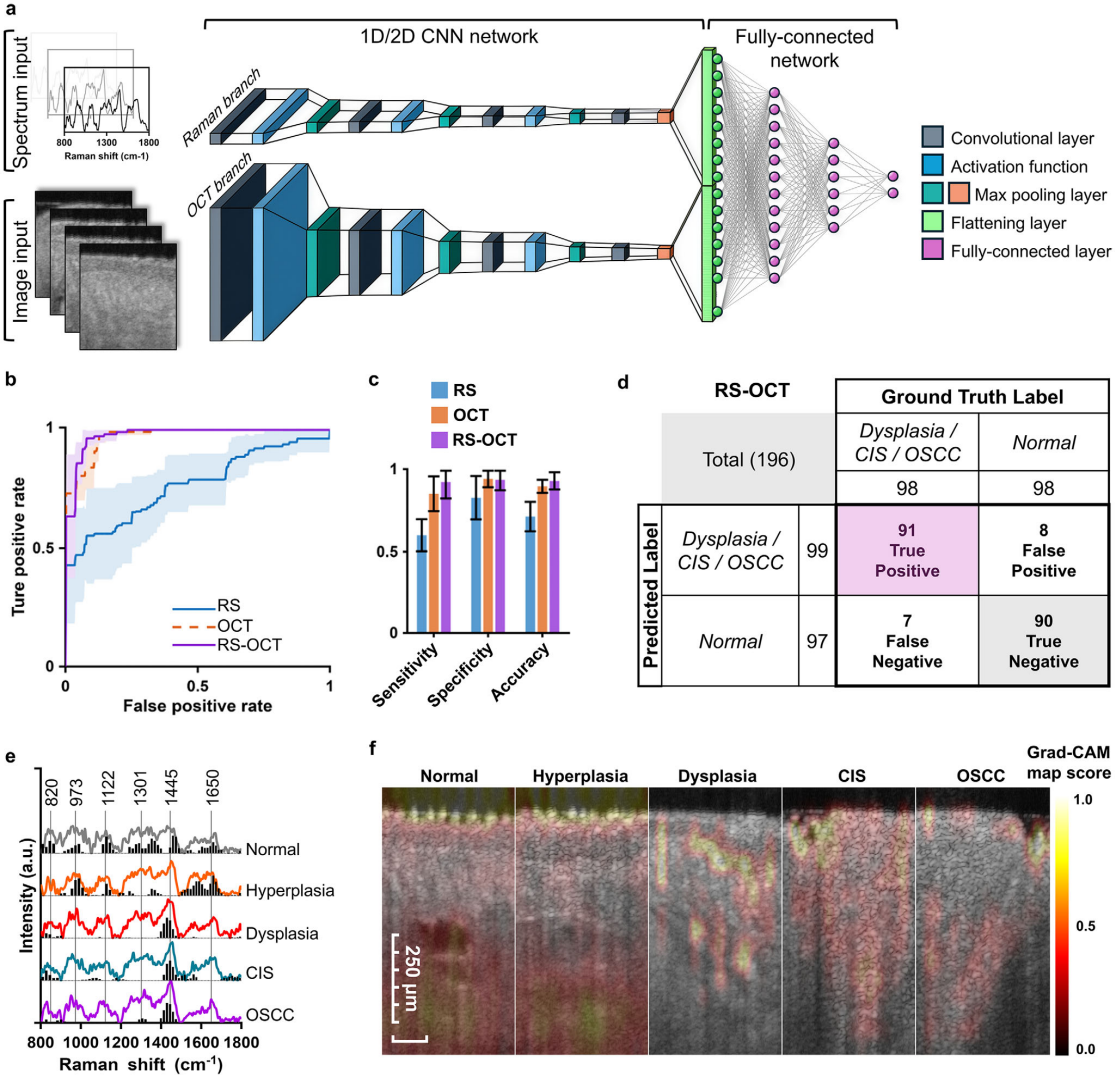
AI‐driven multimodal RS‐OCT data fusion and classification. (a) Deep learning architecture for fusion of Raman spectroscopy (RS) and optical coherence tomography (OCT) data for disease classification. A two‐branch multimodal convolutional neural network (CNN) was developed to independently process spectral and imaging modalities before fusion. The RS branch comprises a 1D convolutional stack with 3 layers (32 to 128 filters) using ReLU activations and a kernel size of 5–15, optimized to extract spectral features across the Raman shift domain. This is followed by a 1D global average pooling layer to reduce dimensionality and capture global spectral patterns. The OCT branch employs a 2D convolutional backbone to extract spatial features from OCT B‐scans, capturing epithelial morphology and microstructural changes. Both branches are concatenated and passed through fully connected layers for joint representation learning, followed by a softmax output layer for classification. (b) Receiver operating characteristic curves for differentiating between disease states using the multimodal RS‐OCT fusion model. The model was trained to distinguish dysplasia and cancer from control tissue samples. (c) Sensitivity, specificity, and accuracy of the fused RS‐OCT model and individual models. (d) Confusion matrix for classification based on fused RS and OCT data, illustrating the model's accuracy and misclassification patterns. (e) Grad‐CAM heatmaps highlighting discriminative spectral features in Raman spectra for control, hyperplasia, dysplasia, CIS and cancer samples. (f) Grad‐CAM visualizations revealing key diagnostic regions in OCT images across control, hyperplasia, dysplasia, CIS and OSCC.

We then investigated the decision‐making process of the AI model to ensure biological plausibility. Gradient‐weighted Class Activation Mapping (Grad‐CAM) was used to visualize the regions within the RS and OCT feature space that most strongly influenced individual classification outcomes (Figure 4e,f). Representative Grad‐CAM overlays were generated for correctly classified samples, revealing clear and modality‐specific activation patterns. For RS inputs, Grad‐CAM showed broad, distributed activations across the Raman spectral range, indicating that the model did not rely on a single peak or narrow feature but instead integrates information from the entire biomolecular fingerprint (Figure 4e). For OCT inputs, Grad‐CAM localized the most salient features within the epithelium across different tissue states. Interestingly, Grad‐CAM captured the filiform papillae structure in normal and hyperplastic tissue, which is known to be effaced in lesions in the 4NQO model. Dysplastic, CIS, and OSCC regions showed strong Grad‐CAM scores near the thickened epithelium areas and particularly in the underlying stroma, related to persisting proliferation above the basal layer and an obscure epithelium‐stroma boundary due to increased epithelial scattering or tumor infiltration, consistent with sites of early pathological remodeling and tumor invasion. Therefore, the results indicate that the network leverages tissue microarchitecture and boundary integrity, key hallmarks used by pathologists, to drive its classification, effectively mimicking human diagnostic reasoning. Together, these findings demonstrate that the network learns representations across modalities, structural cues from OCT and molecular signatures from Raman spectroscopy, with high‐intensity Grad‐CAM regions aligning with biologically and diagnostically meaningful features. The developed fusion model allows for contextualized interpretation, linking molecular alterations to their corresponding architectural changes in vivo.

## 3 Discussion

Current diagnostic methods for HNSCC primarily utilize white‐light endoscopy and biopsy, which provide only intermittent snapshots rather than continuous monitoring. This approach misses the evolving molecular and microscopic changes in tumors over time, making early detection of progression from dysplasia to cancer challenging and potentially delaying crucial treatment decisions. Despite advances in widefield endoscopic imaging, in vivo longitudinal assessment of tissue at both molecular and microstructural levels remains a critical unmet need in oncology.

We present a fully integrated dual‐modality RS‐OCT endoscopic platform that enables real‐time, co‐registered microstructural and biomolecular characterization. This approach allowed dynamic monitoring of tissue changes during carcinogenesis. By leveraging a symmetric fiber geometry and a custom spliced‐optic configuration, we achieved spatial and temporal co‐registration of Raman and OCT signals, ensuring that biomolecular and architectural information is captured from the same tissue location, effectively creating a dynamic snapshot of the tissue state. This opens new avenues for real‐time, in vivo tracking of tumor evolution and response to therapy, with potential to improve how early cancer is detected and monitored.

While the RS signal is primarily derived spatially from the region imaged by OCT, the Raman collection area extends slightly beyond the OCT B‐scan field of view due to the wider spatial sampling of the collection fibers. Based on the fiber geometry, angled excitation/collection optics, and the photon penetration depth at 785 nm in epithelial tissue, we estimate the Raman sensing volume to extend approximately 1 mm laterally and ∼1‐1.5 mm in depth into tissue, broadly overlapping the OCT imaging depth. Future iterations of the platform may incorporate a circular fiber configuration with spiral or Lissajous scanning to enable full en‐face 3D OCT acquisition, thereby improving spatial co‐registration with RS. Interestingly, within our small‐sized dataset, our fiber design shows relatively lower performance for Raman spectroscopy in detecting early lesions. We suspect this is highly dependent on tissue type and epithelial thickness (∼40–80 µm in mice vs ~200‐400 µm in humans), likely because signals from deeper layers dilute the spectral features of superficial lesions. This interpretation is supported by similar probe designs used in human studies, which have achieved accuracies well above 90% [24].

Applying this platform in a longitudinal 4NQO‐induced oral carcinogenesis mouse model, we were able to temporally monitor disease progression from hyperplasia to dysplasia, CIS, and eventually OSCC. OCT imaging captured progressive epithelial thickening and loss of tissue stratification, while RS revealed progressive changes in biochemical composition. The combination of RS and OCT thus enables dynamic tracking of neoplastic transformation, allowing us to not only detect cancerous changes but also to observe the transitional states that precede malignancy. By longitudinally tracking these changes, the platform facilitates a deeper understanding of tumor evolution and heterogeneity, with RS effectively identifying molecular alterations and OCT robustly assessing lesion invasion.

The deep learning fusion model we developed enabled multimodal representation learning, leveraging the microstructural and biomolecular contrast of each modality to enhance diagnostic classification. Grad‐CAM visualizations consistently emphasized structurally relevant tissue regions while not activating on common imaging artifacts such as speckle noise or motion distortions. This indicates that the model was not relying on acquisition‐related artifacts but instead was focusing on meaningful tissue architecture across multiple depths, aligning with established histopathological principles where subsurface changes are critical for identifying dysplasia and cancer. A similar interpretability approach was applied to our RS branch, where importance mapping across the spectral domain revealed that the classifier prioritized specific Raman shifts corresponding to biologically relevant molecular peaks. These findings suggest that the model captures clinically relevant biochemical alterations associated with disease progression. Taken together, these explainability analyses reinforce that the deep learning models are leveraging biologically and clinically interpretable features, structural in OCT and biochemical in Raman spectroscopy, to make accurate predictions. This not only increases confidence in the model's reliability but also provides a bridge between AI‐driven insights and established diagnostic knowledge. A key limitation of the deep learning model is the potential bias introduced by the small dataset. Future work will focus on larger, clinically derived datasets and robust, uncertainty‐aware models to further reduce bias and enhance generalizability. Accordingly, we frame the reported metrics as a feasibility benchmark for real‐time RS‐OCT multimodal fusion in a preclinical animal cohort, not as an estimate of clinical diagnostic accuracy. U, gnder cross‐validation, every animal is evaluated once as an unseen subject, representing the most rigorous use of this limited dataset.

Because OCT acquires images at video rate, far faster than Raman spectroscopy, we can process consecutive B‐frames within the 1 s Raman exposure window. Here we used a simple moving average over 9 frames to reduce speckle noise. A larger averaging window would reduce speckle further but introduces motion blur. More advanced speckle‐reduction algorithms could sharpen the images without sacrificing detail, which would help segment layered tissue; however, whether they would also improve classification accuracy still needs to be tested on a larger dataset.

Robust detection of the epithelium–stroma boundary is difficult because of shadow artifacts, local tissue heterogeneity, and speckle noise. On the tongue, the filiform papillae scatter strongly and cast periodic shadow artifacts onto the tissue beneath them. At the base of each papilla, the epithelium–stroma interface projects upward, which lowers the boundary contrast locally: the lamina propria is less reflective there because the OCT beam strikes it at an oblique angle. Our first‐derivative edge detector finds these high‐gradient boundaries easily, but when it has to bridge a low‐contrast gap, it simply draws the shortest path between the segments it is sure of, rather than the true boundary. For the more difficult cases, such as large lesions, a human reviewer inspected the full video instead of a single frame. Because both the algorithm and these human checks were used to label the initial training set, they set the upper limit on how accurate our boundary detection can be. The averaged stratification metric therefore inherits these boundary errors in low‐contrast regions, and it should therefore be read as an indicator of a transition in tissue state, not as an absolute, quantitative biomarker.

From a broader perspective, this work exemplifies how integrating advanced complementary optical modalities with AI can meaningfully extend our diagnostic capabilities. Its compatibility with existing clinical endoscopes opens avenues not only for oncology, but also for inflammatory and degenerative diseases across the gastrointestinal tract where tissue morphological and molecular changes precede clinical symptoms.

We envision several clinical scenarios in which this platform could be adopted. Both RS and OCT could in principle serve as endoscopic screening modalities, providing rapid, in situ assessment of suspicious lesions. OCT's principal strength lies in evaluating tissue microstructure and, in particular, the depth of invasion, which is critical for staging and treatment planning. The device has the potential to function as a user‐friendly endoscopic diagnostic aid, as it can provide independent probabilistic risk scores directly to the clinician, thereby providing multiple sources of diagnostic information. Future work should focus on establishing robust diagnostic thresholds to distinguish between mild, moderate, and severe dysplasia, CIS, as well as OSCC. Such classification frameworks will likely need to be tailored to specific anatomical sites, given the heterogeneity of tissue characteristics across different organs.

While the developed RS‐OCT platform demonstrates strong potential for real‐time morphomolecular tissue characterization, several limitations should be considered in the context of clinical translation. From a practical perspective, the system offers key advantages including label‐free operation, integration with standard endoscopic workflows, and the ability to provide combined structural and molecular information in real‐time. However, these benefits are balanced by challenges related to system complexity and the cost associated with multimodal instrumentation, which may impact adoption in routine clinical settings. A key methodological limitation lies in the temporal co‐registration of RS and OCT, given their inherently different acquisition rates (1 Hz vs. video‐rate). The current approach, based on random OCT frame selection within each RS acquisition window, assumes short‐term tissue stability and was chosen for its simplicity and robustness in exploratory studies. Nevertheless, this strategy may introduce temporal mismatch and limit the ability to capture rapid dynamic changes, potentially affecting the precision of temporal morphomolecular tracking. Future developments will focus on tighter hardware‐level synchronization and advanced data fusion strategies to improve temporal alignment.

A limitation of this study is that, although the RS‐OCT probes were designed with future integration into flexible endoscopic systems in mind (outer diameters 2.2 and 3.4 mm, compatible with typical endoscope working channels), direct endoscopic deployment was not performed. All measurements were acquired on externally positioned mouse tongues to ensure stable and reproducible multimodal data acquisition in this small animal model. While this enabled controlled imaging and reliable histopathological correlation, it does not fully replicate the mechanical constraints, motion artifacts, and clinical workflow of in vivo endoscopy. Among these, bulk tissue motion is likely the foremost obstacle to in vivo endoscopic translation: cardiac, respiratory, and peristaltic movement, together with operator‐induced probe motion, can blur OCT B‐scans and corrupt the 1 s Raman integration that underpins co‐registration. We observed a dominant cardiac motion in OCT videos and a minor motion blur on individual images. This motion was largely reduced if the probe was in good contact with the tissue. Mitigating this will require hardware‐ or image‐based motion tracking and gating, and deep‐learning denoising and registration, which we regard as essential next steps before clinical deployment.

Looking ahead, we envision the integration of this dual‐modality system with additional contrast mechanisms such as photoacoustic imaging, multi‐photon imaging, or fluorescence lifetime imaging to capture microvascular, functional, and metabolic cues in parallel under widefield guidance. We expect such advances to be realized with the use of photonic crystal fibres. Correlation with ex vivo spatial biology assists in explainability of Raman data [25]. Further pragmatic developments could leverage AI‐enhanced signal reconstruction, wherein RS clinical acquisition <100 ms will be possible [26, 27]. As optical technologies converge with computational advances, multimodal endoscopy such as RS‐OCT could provide earlier, more comprehensive and more temporally resolved insight into disease state transitions and trajectory.

## 4 Conclusion

We developed a compact, unified endoscopic platform that integrates RS and OCT within a miniaturized, forward‐viewing fiber‐optic probe, enabling real‐time, co‐registered molecular and structural imaging in vivo. By combining subsecond RS acquisition with video‐rate OCT and distal scanning, the system achieves high spatial and biomolecular resolution within a single optical field. In a longitudinal HNSCC mouse model, it captured epithelial remodeling alongside the molecular changes underlying malignant progression. This multimodal approach overcomes the limitations of individual modalities, providing a more comprehensive and quantitative view of disease evolution. By linking tissue morphology with intrinsic molecular contrast, the platform shows promise for improving early cancer detection and guiding precision interventions during endoscopy. With the addition of AI‐driven analysis and real‐time data fusion, such systems could advance toward a true optical biopsy paradigm, supporting earlier detection, more accurate diagnosis, and personalized treatment strategies in oncology. Although OCT carried much of the structural discriminative power in this thin murine epithelium, the central advance is the synergistic, explainable fusion of structure and biochemistry: RS contributes molecular specificity that OCT cannot access, and Grad‐CAM confirms that classification draws on biologically meaningful spectral and architectural features. We anticipate a larger molecular contribution from RS in human mucosa, where the epithelium is several‐fold thicker.

## 5 Materials and Methods

### 5.1 RS‐OCT Instrumentation and Fiber‐Optic Probe Fabrication

The RS‐OCT instrumentation (Figure 1a) was based on a 2D spectral domain OCT scanning platform and an in‐house developed Raman system. For OCT, we used a 1325 nm superluminescent light emitting diode (SLED) (SLD1325, Thorlabs, Inc: 1325 nm/ 10 mW/ 100 nm), a short‐wave infrared Cobra 1300 spectrometer (C1300‐1310/150, Wasatch Photonics, 147 kHz max line rate) and a fiber polarization controller (FPC032, Thorlabs, Inc). The spectrometer was interfaced to a Camera Link image acquisition device (PCIe‐1433, National Instruments Corp.). A visible laser (488 nm Toptica Ibeam Smart) was coupled into the same light path of the OCT for alignment to identify the location of the OCT output beam. The acquisition of OCT frames was synchronized to the beam scanning system using an analog output device (USB‐6343, National Instruments). We used a 785 nm diode laser (Cleanlaze 450 mW, B&W Tek) coupled via a 200 µm fiber (FG200LEA, Thorlabs, Inc.) for RS. The RS system comprised a high‐throughput spectrometer (Acton LS785, Teledyne) with a thermoelectric‐cooled deep depletion charge‐coupled device (CCD) (Pixis 1340 × 400, Teledyne). The spectrometer had no slit, so that the linear array of input fibers (FG105LCA, Thorlabs, Inc.) acted as the slit.

The fiber‐optic probe was constructed in‐house in a dedicated facility using motorized precision (< 5 µm) assembly (Figures 1 and S1). The fiber‐probe employed a layered fiber arrangement resembling a sandwich, with two RS collection/excitation fiber arrays positioned on either side of a centrally aligned single‐mode fiber (SMF‐28 Ultra, Corning Inc.) dedicated to OCT. These RS fibers were angled slightly inward toward the optical axis (Figure S1b). This configuration facilitated OCT B‐scanning through a central slit, achieving a 1 mm field of view and delivering axial and transverse resolutions of 10 and ∼25 µm, respectively (Figures 1a and S1c). The symmetrical layout preserved a fixed spatial relationship between the structural (OCT) and molecular (RS) sensing elements, enabling accurate alignment of morphological structures with their corresponding biochemical markers. Since RS is incompatible with distal quartz lenses, the OCT focusing optics were developed by splicing a NCF (FG125LA, Thorlabs, Inc.) and GIF (GIF625, Thorlabs, Inc.) fiber to the cantilever single‐mode fiber (Figure 1c). We developed both a 3.4 and 2.2 mm diameter fiber probe.

To assemble the dual‐modality probe, a piezo tube with four electrodes was first mounted to a stainless‐steel tube (Figure S1a). The piezo tube was connected to a voltage source via four thin wires and secured to the stainless‐steel tube using a piezo holder. The motion of the free end of the 0.45 mm (for 2.2 mm version probe) or 2.2 mm (for 3.4 mm version probe) piezo tube (Physik Instrumente) was controllable by voltage. Next, ten 200 µm multimode low‐OH silica fibers (FG200LEA, Thorlabs, Inc.) for Raman excitation and collection were cleaved flat and grouped into two linear fiber arrays, each containing five fibers (Figure S1b). These were deposited with thin‐film bandpass/longpass filters (Shenzhen Photonstream Ltd). The linear fiber bundles were mounted onto a holder with a wedge design, forcing the two bundles to bend inward to increase the overlapping volume of excitation and collection with OCT single‐mode fiber. In the third step, a CaF_2_ window was attached to the end facet of the linear fiber bundle holder. The holder was angled at the end facet to mitigate OCT back reflection (Figure S1c). The CaF_2_ window sealed the probe and prevented contamination. Once the window was attached, an OCT fiber, consisting of a single‐mode fiber and distal fiber optics, was gently inserted through the inner hole of the piezo tube using motorized precision assembly. The OCT fiber was positioned close to the window (∼150 µm) due to its limited working distance. Alignment of the OCT fiber was performed under a microscope for lateral positioning and using OCT imaging for axial positioning. Once aligned, the OCT fiber was secured to the free end of the piezo tube using epoxy adhesive (Figure S1d,g). Finally, the proximal ends of the fibers were interfaced with the RS‐OCT system. The clinically relevant OCT FOV of 1 mm is approximately equivalent to, or slightly smaller than, the size of biopsies. For the RS fibers, the central fibers of the two linear fiber bundles were used for excitation, where the Raman laser light is coupled into the fibers. The remaining Raman fibers were grouped into a round fiber bundle and connected to the spectrometer via inline bandpass and longpass filters (BLP01‐785R‐25 & LL01‐785‐25, Semrock).

### 5.2 RS‐OCT Real‐Time Acquisition Framework

We developed an acquisition framework in Python. The software enables real‐time RS and OCT data acquisition and analysis (Figure S2). This comprehensive real‐time software package manages the fiber scan trajectory, generates synchronization signals, reads the OCT B‐scan frames and Raman spectra, and preprocesses the data. The fiber scanner and OCT spectrometer are hardware‐synchronized to ensure precise image construction, while the Raman spectrometer is software‐synchronized with the OCT spectrometer, allowing for temporal co‐registration of the two modalities. Since the Raman spectrometer operates much more slowly than the OCT spectrometer, a multithreading design is employed to run the fast OCT data acquisition in parallel with the slower Raman acquisition. Raman data preprocessing includes simple dark current noise subtraction and intensity scaling. In contrast, OCT data preprocessing is more complex, involving background removal, k‐domain linearization, FFT, and normalization. These OCT preprocessing tasks are offloaded to the GPU, enabling low‐latency, video‐rate rendering of the OCT images.

### 5.3 Preprocessing and Analysis of Raman Data

The obtained spectra undergo wavelength calibration using an atomic lamp (Ocean Optics HG‐1). The background Raman spectrum of the system was subtracted from the tissue Raman spectra. To eliminate autofluorescence background, a custom fifth‐order polynomial fit function [9], constrained to the lower segment of the Raman spectrum, was applied. Finally, the spectra were normalized to their integrated area to enable relative abundance estimation and reduce absolute intensity fluctuations.

PCA was then applied to reduce dimensionality and identify dominant sources of spectral variance. The number of PCs retained for downstream analysis was determined based on the cumulative variance explained and the inspection of scree plots. PCs that collectively captured >98% of the total variance were initially considered. Individual components were further evaluated for their relevance to tissue pathology by inspecting loadings and their correlation with histopathological classifications. This approach ensured that retained PCs represented meaningful biochemical variation rather than noise.

### 5.4 OCT Image Processing and Quantitative Morphological Feature Extraction

To extract morphometric parameters relevant to epithelial architecture and tissue organization, we developed a quantitative OCT image analysis pipeline that processes B‐scan images to derive two key metrics: epithelial thickness and an epithelial stratification/disorganization index. These features were selected based on their clinical relevance to dysplasia and neoplastic progression in mucosal tissues. Raw OCT B‐scans were first normalized to the fiber tip reflection (that appears as a horizontal bright line at the top of the supplementary OCT video) to compensate for light source fluctuations. To mitigate sensitivity roll‐off due to the finite size of the CCD pixels, depth‐dependent normalization was applied by dividing each A‐line by a calibrated sensitivity curve. A moving average of 9 consecutive frames, gated by a separate motion detector based on decorrelation, was applied to reduce speckle noise and minimize motion blur. Following preprocessing, image segmentation was performed to delineate the epithelial surface and basement membrane. A semi‐automated approach was employed to segment the epithelial layer:

1. The epithelial surface was identified as the first significant intensity rise along each A‐line (first derivative Dijkstra's‐shortest‐path‐based edge detection).
2. The stroma surface was defined as the second significant intensity rise below the epithelial surface, reflecting the transition between the epithelium and lamina propria/muscle. This was automated by a ResNet‐50 model trained on a manually labeled dataset and a first derivative edge detection.

Epithelial thickness was computed per A‐line as the Euclidean distance between the epithelial surface and basement membrane along the axial direction. The final value for each OCT B‐scan was derived by averaging thickness measurements across all valid A‐lines (typically 256 per frame after downsampling). This provided a robust representation of overall epithelial thickening, a hallmark of dysplasia and malignancy.

To quantify epithelial architectural organization, we defined a stratification index based on the variance in OCT intensity between the epithelium and stroma. For each A‐line, the tissue depth was divided into two zones of equal thickness: a 21 µm lower epithelium region and a 21 µm upper stroma region. The zone thickness of 21 µm was chosen as a balance between two constraints: the minimum epithelial thickness in mouse tongues is approximately 42 µm, which sets the upper bound, and the OCT axial resolution is about 10 µm, which sets the lower bound. Selecting a midpoint value ensures that each zone is thick enough to capture relevant tissue structure while remaining within the resolution limits of the imaging system. We calculated the mean pixel intensities within these two zones for each A‐line and computed a normalized stratification index as: $SI=-20\log(I_{e}/I_{s})$, where *I_e_* is the mean intensity of the lower epithelium and *I_s_* is the mean intensity of the upper stroma. In well‐organized (healthy) epithelium, stratification results in consistent reflectivity gradients between the basal layers and the underlying stroma/muscle, yielding predictable SI values. In contrast, dysplastic or disorganized epithelium exhibits disrupted polarity and reflectivity patterns, leading to altered SI ratios. All morphological features (mean epithelial thickness and stratification index) were computed for each imaging session per animal and temporally tracked across timepoints. These features were subsequently aligned with molecular (RS) features and endpoint histopathology labels for statistical analysis and multimodal classification.

### 5.5 Temporal Co‐Registration of OCT and RS

Analyzing large volumes of multimodal data acquired at different temporal resolutions poses a significant challenge, especially in ensuring accurate alignment and integration across modalities. In our study, we combined RS, which provides one‐dimensional spectral data at a rate of one spectrum per second, with OCT, which produces two‐dimensional imaging at a high video frame rate of 46 frames per second. To align these temporally mismatched datasets, we implemented a temporal co‐registration approach. Specifically, for each RS spectral acquisition (1 Hz), we randomly selected one of the corresponding OCT frames captured within the same 1 s interval. This method assumes that all OCT frames within that second are sufficiently similar for the purposes of co‐registration. We chose this approach for its simplicity and practicality in exploratory data analysis. It allows efficient pairing of OCT and RS data without the need for complex synchronization algorithms or hardware‐based triggers.

### 5.6 AI‐Based Multimodal Fusion and Classification

To enable classification from limited in vivo data, we implemented a deep learning framework for the fusion of RS and OCT data using a multimodal CNN. The goal was to learn a shared representation that captures the synergistic diagnostic value of both biochemical and morphological features. The raw dataset consisted of *n* = 196 Raman spectra and *n* = 196 OCT B‐scan frames, each co‐registered and acquired from temporally tracked tissue sites across 11 mice. All RS and OCT data were synchronized temporally and spatially via acquisition timestamps and scanner positional metadata. Details of the dataset have been outlined in Figure S6. Owing to the limited in vivo dataset, the classifier was evaluated with animal‐independent fivefold cross‐validation across the 11 mice contributing co‐registered RS‐OCT data. Each fold withheld all measurements from two held‐out mice (typically one 4NQO‐treated and one control). Because the cohort of 11 does not divide evenly, a single fold withheld three mice. Every animal was therefore used for testing exactly once and never appeared in both the training and test sets. Reported benchmarks (reciever operating characteristic, accuracy, sensitivity, and specificity) were aggregated measures from the cross‐validation due to limited data. The network ingested only preprocessed data: background‐subtracted, autofluorescence‐corrected, and vector‐normalized Raman spectra, and background‐removed, k‐linearized, FFT‐reconstructed, normalized, and despeckled OCT B‐scan images. No raw data and none of the hand‐engineered morphological features (epithelial thickness, stratification index) were used as network inputs; those engineered features served exclusively for the quantitative and correlation analyses presented in Figure 3.

We designed a two‐branch multimodal CNN to independently process Raman and OCT data streams before joint fusion and classification. The Raman branch consisted of a 1D convolutional stack (3 layers, 32–128 filters, ReLU activations, kernel size of 5–15), followed by a 1D global average pooling layer. The OCT branch was implemented using a 2D convolutional backbone (based on a truncated ResNet‐18 architecture), optimized for OCT's texture‐rich features. Both modality‐specific feature embeddings were concatenated and passed through a series of fully connected layers (FC1: 64 nodes, FC2: 64 nodes) before a softmax output layer using a weighted classification layer with categorical cross‐entropy loss and softmax activation to address class imbalance. Optimization was performed using the Adam optimizer (learning rate = 1e–4, full‐batch training), with early stopping as an essential component for such a small dataset to choose the optimal model based on validation loss plateauing after 100 epochs. Model performance was evaluated only on the held‐out test set using standard classification metrics, including accuracy, sensitivity, specificity, and area under the receiver operating characteristic curve (AUC). Pooled metrics were computed across all held‐out animals and therefore weighted larger folds more heavily. Fold means were unweighted. To assess the contribution of each modality, we deactivated either the RS or OCT branch, allowing us to quantify the added diagnostic value of multimodal fusion. All models were implemented in MATLAB (v2023) and trained using an NVIDIA RTX A6000 GPU. Training took a few minutes per fold, and all results were aggregated over cross‐validation folds.

### 5.7 Modal Specific Interpretability With Grad‐CAM

To interpret the modality‐specific contributions to the final classification, Grad‐CAM was applied independently to each branch of the multimodal CNN. For the Raman branch, gradients of the output class score were computed with respect to the final 1D convolutional layer. These were used to generate a weighted activation map highlighting key spectral regions influencing the model's decision. For the OCT branch, Grad‐CAM was applied to the last convolutional block of the truncated ResNet‐18 backbone. The resulting 2D heatmaps identified spatial regions within OCT images most relevant to the classification. By isolating Grad‐CAM computation to each branch before fusion, we obtained interpretable, modality‐specific visualizations of learned discriminative features.

### 5.8 4NQO Murine Carcinogenesis Model

All animal work was approved locally at King's College London (UK) and performed under a UK Government Home Office Project License (PP0313918). The animal experiment was designed with careful consideration of the 3Rs (Replacement, Reduction, and Refinement) [28]. C57BL/6 mice were treated with 4‐Nitroquinoline N‐oxide (4NQO) diluted to 100 µg/mL in drinking water for 16 weeks. 4NQO models are known to replicate the alterations caused by tobacco mutagens, in which the disease progresses through a series of premalignant stages and exhibits histological features that closely resemble human HNSCC [7]. Exposure to 4NQO leads to the development of multifocal dysplastic lesions in 100% of the 4NQO‐treated mice. Of these, ∼50% will develop into invasive cancer within 22 weeks. 4NQO‐containing water was prepared and changed once a week for 16 weeks. During the experiments, the mice were maintained with regular mouse chow and water (with or without 4NQO) ad libitum (*n* = 10 control and *n* = 10 4NQO‐treated group). After that period, mice were given normal drinking water until the endpoint of 22 weeks or until mice lost > 15% of their maximum body weight. Over multiple time points, 4NQO‐treated and control mice were sedated with inhaled isoflurane, and the oral cavities were screened for any visible lesions and RS‐OCT data measurements.

### 5.9 Histology

At the experimental endpoint, mice were euthanized in accordance with institutional animal care and use protocols. Whole tongues were carefully resected from the oral cavity using sterile surgical instruments. Care was taken to preserve both the tumor‐bearing and surrounding non‐tumorous mucosa for comprehensive histological evaluation. For frozen sections, tongue tissues were embedded in optimal cutting temperature compound, cryosectioned, and stained with hematoxylin and eosin (H&E) by conventional methods. H&E slides were scanned with the NanoZoomer 2.0RS Digital Slide Scanner (Hamamatsu Photonics K.K., Japan) with 0.23 µm/pixel, 40 × high resolution (Brightfield) mode.

Histopathological evaluation was performed using predefined and standardized diagnostic criteria based on the WHO Classification of Head and Neck Tumours (Fourth Edition) and established histopathological guidelines. All assessments were conducted in a blinded manner with respect to spectral analysis. Lesions were systematically classified into hyperplasia, dysplasia (graded as mild, moderate, or severe based on the extent of epithelial involvement and degree of cytologic atypia), CIS (high‐grade squamous intraepithelial neoplasia), and invasive OSCC, based on architectural and cytological features, as well as the presence or absence of invasion into surrounding tissue. Lesions were classified as squamous proliferative lesions or squamous intraepithelial neoplasia in the 4NQO model when they did not meet the diagnostic criteria for CIS, specifically demonstrating residual epithelial maturation, incomplete full‐thickness atypia, and focal preservation of stratification.

### 5.10 In Vivo RS‐OCT Imaging of Mice

For in vivo imaging, both 4NQO‐treated and control mice were placed on a heating pad and sedated with inhaled isoflurane. The tongue was gently pulled out with tweezers, and the RS‐OCT probe was gently positioned in direct contact with the mucosal surface at multiple predefined sites across the tongue (a single anatomical site). To ensure consistent and reproducible sampling, probe placement was guided by anatomical landmarks and maintained using a micromanipulator arm. Raman spectra were acquired with an integration time of 1.0 s per location, while OCT imaging was performed continuously at a video‐rate acquisition speed, enabling structural assessment in real‐time. A foot pedal was utilized to control whether to save the data to disk. The optical power delivered to the tissue was 7 mW for the 1325 nm OCT channel and 60 mW for the 785 nm Raman excitation. To achieve temporal and spatial co‐registration of Raman and OCT measurements, data acquisition was synchronized through a custom software interface that triggered simultaneous frame capture and spectral collection at each site, where the timestamp for each frame/spectrum acquisition was recorded. This ensured precise pairing of morphological and molecular data from the same tissue region across all imaging sessions. Repeated measurements were performed longitudinally at identical sites across time points (weeks 10–20) to enable morphomolecular tracking of carcinogenic progression in vivo. Lesion sizes were approximately 1–2 mm at early time points and increased to ∼2–4 mm at later stages. The 3D field of view for each RS‐OCT acquisition was approximately 0.2 mm × 1 mm × 1 mm (width × length × depth). To ensure label quality, we included only RS‐OCT measurements that formed confidently co‐registered pairs with matched histopathological ground truth; this required lesions to be accessible to the forward‐viewing probe (excluding the posterior tongue) and visible for confirmatory sampling, yielding 11 of the 20 mice for classification.

### 5.11 Statistical Analysis

Data are presented as individual measurements or mean ± standard deviation (SD), as specified in the figure legends. Between‐group comparisons used two‐sided Wilcoxon rank‐sum tests with Benjamini–Hochberg false‐discovery‐rate (BH‐FDR) correction; adjusted *p* < 0.05 was considered significant.

## Author Contributions

**J.Q**. designed the study, performed the experiments, interpreted and analyzed the data, generated the figures, and wrote the manuscript. **P.G.B**. designed and performed animal experiments, histology, contributed to scientific discussion, and data analysis. **V.K**. contributed to experiments. **J.G**. performed histology. **S.L**. contributed to histology. **C.C**. contributed to scientific discussion and data interpretation. **R.J.C**. contributed to scientific discussion and data interpretation. **M.S.B**. designed and supervised the study, interpreted and analyzed the data, and wrote the manuscript.

## Code availability

The software used in this study is not publicly available. However, requests for code access for academic research purposes can be directed to the corresponding author.

## Conflicts of Interest

M.S. Bergholt, J. Qiu, and R. J. Cook have filed a provisional patent application. The remaining authors declare no conflicts of interest.

## Supporting information




**Supporting File 1**: advs77613‐sup‐0001‐SuppMat.docx.

**Supporting File 2**: advs77613‐sup‐0002‐VideoS1.mp4.

## Data Availability

The data that support the findings of this study are available from the corresponding author upon reasonable request.
