# Supplementary material for: Longitudinal Morphomolecular Monitoring of Head and Neck Carcinogenesis
Source: Adv Sci (Weinh). 2026 Sep 30:e22344. Online ahead of print. doi: 10.1002/advs.202522344 (PMC13625584; doi:10.1002/advs.202522344)
Supplement: Supplementary file 1 [file ADVS-9999-e22344-s002.docx]

**Supplementary information**

**Longitudinal Morphomolecular Monitoring of Head and Neck Carcinogenesis**

Jianrong Qiu^1^, Priyanka G. Bhosale^1,2^, Vishal Kumar^1^, Jeyrroy Gabriel^3,4^, Shiyue Liu^1^, Ciro Chiappini^1^, Richard J. Cook^5^, and Mads S. Bergholt^1,^*

*^1^Centre for Craniofacial & Regenerative Biology, King’s College London, SE1 9RT London, United Kingdom*

*^2^Department of Haematology, University College London Cancer Institute, WC1E 6DD, London,*

*United Kingdom*

*^3^CGRM, BMBS, Guy’s Campus, King’s College London, SE1 9RT, London, United Kingdom*

*^4^St. John’s Histopathology, Tissue Sciences, Synnovis Analytics, St. Thomas’ Hospital, London, United Kingdom*

*^5^Centre for Oral Clinical and Translational Sciences, King’s College London, SE1 9RT London, United Kingdom*

** Corresponding author*: [mads.bergholt@kcl.ac.uk](mailto:mads.bergholt@kcl.ac.uk)

Keywords: Raman spectroscopy, optical coherence tomography, fiber-optics, endoscopy, artificial intelligence

**This PDF file includes:**

**Supplementary Figures**

**Supplementary Fig. S1.**

**Supplementary Fig. S2.**

**Supplementary Fig. S3.**

**Supplementary Fig. S4.**

**Supplementary Fig. S5.**

**Supplementary Fig. S6.**

**Supplementary Fig. S7.**

**Fig. S1: Design and assembly of the integrated fiber-optic RS-OCT probe.** Schematic diagrams (not to scale) show cross-sectional and longitudinal views of the distal and proximal ends. The probe integrates multiple fibers for co-localized optical coherence tomography (OCT) and Raman spectroscopy (RS). **a**, A four-segmented piezo tube scanner was securely mounted in a stainless-steel tube. **b**, A miniaturized, sandwich-like configuration positions two angled RS excitation/collection fiber arrays on either side of the central OCT fiber. **c**, Optical components are fixed with epoxy and a custom fiber-bundle holder, and a CaF₂ window seals the distal tip to provide optical access and biocompatibility. **d**, The distal tip contains a central single-mode fiber for OCT beam delivery, surrounded by ten multimode fibers, two for RS excitation and eight for RS collection, within the stainless-steel tube. **e-h**, Microscopy images confirm precise fiber alignment. Labels indicate structural materials and optical elements used in fabrication.

**Fig. S2: Schematic of the integrated data acquisition and processing framework for co-registered RS-OCT measurements.** A parallelized workflow enables simultaneous acquisition and processing of Raman spectroscopy (RS) and optical coherence tomography (OCT) data. OCT images are firstly processed using a GPU-based pipeline comprising background removal, k-domain linearization, Fast Fourier Transform (FFT), offset/scaling for high-resolution reconstruction, and are then processed with light-source fluctuation correction, sensitivity roll-off correction and speckle noise reduction. Raman spectra are processed on the CPU via background subtraction, autofluorescence removal by polynomial fitting, and vector normalization. Temporal co-registration is achieved by synchronizing each one-second RS acquisition with a randomly selected OCT frame from a 46-fps sequence, ensuring morphological (OCT) and molecular (RS) data correspond to the same tissue state.

Fig. S3: **Survival plot and pathology outcome of mice in the 4NQO-induced oral carcinogenesis model.** **a,** Kaplan-Meier plots showing the survival probability of mice over the treatment and *in vivo* RS-OCT acquisition period in treated and control group (n=10 per group). Statistical test used: log-rank test (*p < 0.001)*. The 4NQO-treated mice were sacrificed depending on their health conditions, typically, within 6 weeks after the optical monitoring. **b,** Endpoint histological outcome (percentage of mice with dysplasia, carcinoma *in situ* (CIS), papilloma, microinvasive and OSCC. **c,** Endpoint histological outcome showing the number of lesions per mouse with dysplasia, CIS, papilloma, microinvasive and OSCC. d, Macroscopic photos of resected tissues following 4NQO exposure illustrate the heterogeneous presentation of oral lesions. Also shown is a physical ruler. The mouse tongue width is approximately 4 mm. The gross morphological changes, visible as distinct lesions, correspond to a range of histological features, including dysplasia, CIS, papilloma, microinvasive and invasive OSCC. **e**, A photo taken in an *in-vivo* measurement shows how the RS-OCT probe was maneuvered for data collection. A tweezer was used to pull out the tongue from the anesthetized mouse by one trained operator. The sides, up surface, or the tip of the tongue was selectively exposed by changing the tweezer position and the pulling direction. **f**, Far-field round spot (red) of one Raman fiber and line spot (cyan) of scanning OCT fiber. The cyan spot is produced by the aiming laser and indicates the position of the otherwise invisible OCT beam. The offset between the Raman and OCT beams arises primarily from the tilt of the Raman fibre.

**Fig. S4: Additional representative H&E and OCT data supporting the main findings.** Extended H&E-stained sections from control and 4NQO-treated tissue show normal epithelium, hyperplasia, and oral lesions at various stages, including dysplasia of different grades, carcinoma *in situ* (CIS)-like squamous proliferative lesions, and invasive OSCC. Additional *in vivo* OCT images from multiple animals and time points consistently reveal hallmark features of epithelial transformation, thickening, increased scattering, and loss of tissue organization. Scale bars are shown on the OCT and histology panels.

**Fig. S5: Raman difference spectra highlighting molecular changes during oral carcinogenesis.** Mean difference Raman spectra (±1 standard deviation) are shown for key pathological transitions in the 4NQO-induced murine model: (hyperplasia – normal), (dysplasia – hyperplasia), (CIS – dysplasia) and (OSCC – CIS). Each spectrum depicts the mean normalized intensity difference between adjacent histological stages, revealing progressive biochemical alterations in tissue composition.

**Fig. S6: Overview of the dataset for network training and test. a**, A flow chart shows how the data were labeled. Co-registered image-spectrum pairs from the last three timepoints were considered. Data with “Lesion” mark, which indicates data collection on visible lesions, were selected for lesion subtype confirmation. With an established library of H&E images of the whole tongue, the lesion was paired with a few adjacent microscopic slides based on the geometry. Successful paired lesions were then categorized into dysplasia, carcinoma *in situ* like squamous proliferative lesions, and OSCC. For data without a “Lesion” mark and data collected from control mice, OCT images were inspected by human to remove a small portion of outliers with distinct appearance to the majority part. **b**, Lesions confirmed with histology were distributed among six 4NQO-treated mice, where 98 spectrum-image pairs were collected from 8 independent tissue sites or 11 tissue sites if measurements from the same tissue site at different time points were considered independent. **c**, Histopathological results of the lesions under test. For the deep-learning classification, 5-fold cross-validation (mice independent) was used; lesions from the same animal therefore never appear simultaneously in training and test, and the aggregate metrics reflect evaluation of all animals as unseen subjects.

**Fig. S7: Benchmarking performance of individual and fused modalities in the AI-enabled RS-OCT classification framework on various cross validation strategies.** (**a-c**) Performance benchmarks (ROC, sensitivity, specificity, accuracy and confusion table) using leave-one-tissue-site-out cross-validation. (**d**) Comparisons of n = 5-fold (mice independent) versus leave-one-tissue-site-out cross-validation, fold mean with 95% confidence interval (CI).
